# An Internet-Based Survey of Influenza Vaccination Coverage in Healthcare Workers in China, 2018/2019 Season

**DOI:** 10.3390/vaccines8010006

**Published:** 2019-12-26

**Authors:** Haitao Liu, Yayun Tan, Muli Zhang, Zhibin Peng, Jiandong Zheng, Ying Qin, Zhiqiang Guo, Junhua Yao, Fen Pang, Teng Ma, Wenjing Duan, Zhongjie Li, Luzhao Feng, Mo Hao

**Affiliations:** 1 Research Institute of Health Development Strategies & Collaborative Innovation Center of Social Risks Governance in Health, Fudan University, Shanghai 200032, China; 2 Suzhou Center for Disease Control and Prevention, Suzhou 215000, China; 3 Key Laboratory of Surveillance and Early-Warning on Infectious Disease, Division of Infectious Disease, Chinese Center for Disease Control and Prevention, Beijing 102206, China; 4 Beijing Dingxiangyuan Tiantian Health Technology, Beijing 100020, China

**Keywords:** influenza, healthcare workers, vaccination coverage, internet-based survey, China

## Abstract

Influenza vaccination coverage was low among healthcare workers (HCWs) in China. In October 2018, the National Health Commission of China began to require all hospitals to provide free influenza vaccination for HCWs to increase vaccine uptake, and no study on vaccine coverage among HCWs at the national level after the announcement of new policy. This evaluation aims to investigate self-reported influenza vaccination coverage among HCWs and factors that may affect vaccine receipt during the 2018/2019 influenza season. We delivered an opt-in internet panel survey among registered HCWs of DXY forum (the biggest online forum for HCWs in China). The survey was self-administered using a standard questionnaire to collect information on demographics, occupational characteristics, policy implementation, influenza vaccination and influence factors. We conducted multivariate logistic regression analysis to assess factors associated with receipt of influenza vaccine. The response rate of this online survey was 3.6%. The seasonal influenza vaccine coverage reported among HCWs surveyed during the 2018/2019 season was 11.6% (472/4078). Only 19.0% (774/4078) of HCWs surveyed reported free policy in their workplace. Combing free policy and workplace requirement proved to be effective to improve influenza vaccination coverage in HCWs (PR = 6.90, 95% CI: 6.03–7.65). The influenza vaccination coverage among surveyed HCWs in China was low during the 2018/2019 season. To increase future vaccination uptake, we recommend a multi-faceted strategy that include free policy, workplace requirement and promotion, on-site vaccination, and monitoring.

## 1. Introduction

Seasonal influenza is estimated to cause 88,100 respiratory deaths annually in China [1]. Although influenza vaccination is the most effective way to prevent influenza infection [2], vaccination coverage in China was estimated to be 1.5–2.2% among the general population during 2004 and 2014 [3]. Healthcare workers (HCWs) are one of the recommended groups for influenza vaccination because of their risk of getting infected and vaccination of HCWs significantly reduced influenza-like illness and all-cause mortality among patients, as well as number of working days saved [4,5]. Regional estimates of influenza vaccination coverage among Chinese HCWs have ranged from 5–18% [6,7], but data describe national coverage estimates was lacked.

Influenza vaccine is generally paid for out-of-pocket in China and there is no immunization program for influenza nationally. In October 2018, China’s National Health Commission issued an official document, requiring all hospitals to provide free influenza vaccination for HCWs [8]. However, the status of the implementation of this policy and its effect on influenza vaccination coverage is unknown. We aimed to investigate the 2018/2019 influenza vaccination coverage, factors associated with influenza vaccination, including workplace vaccination policies, and reasons for influenza vaccine receipt or non-receipt among HCWs.

## 2. Materials and Methods

### 2.1. Study Design

We conducted an opt-in internet based cross-sectional study from 21 March to 15 April 2019, and enrolled HCWs registered on DXY forum. DXY is an online forum for HCWs, medical researchers, teachers, and students. There were approximately 2.3 million registered HCWs, accounting for about 20% of all the HCWs in mainland China [9]. Our eligibility criteria for participation in the survey required that users list an occupation as doctor, nurse, radiologist, pharmacist, medical technician, administrative support staff, and confirmed their workplace as a hospital. We randomly sampled a proportion of those eligible for participation, and sent them an email invitation letter that included a link to a standardized questionnaire for completion.

We used a simple random sample formula to calculate sample size. Assuming a predicted influenza vaccine coverage among HCWs of 10% with an allowable error of 1%, we calculated a sample size of 3445. To allow for disqualification of incomplete questionnaires, we increased the sample size by 10%, with a final target sample population of 3789.

### 2.2. Data Collection

Participants were required to complete a standardized online questionnaire. Data were collected on healthcare workers demographic and occupational characteristics, self-reported influenza vaccination status during the 2018/2019 influenza season, workplace policies related to influenza vaccination, and reasons for receipt or non-receipt. Respondents were able to select more than one reason and no rank order was required.

Based on the National Influenza Prevention and Control Scheme (pilot version) from China’s National Health Commission, we classified respondents who worked in respiratory disease, infectious disease, pediatrics, gynecology, and obstetrics and emergency departments as working in a “high risk department” [8].

For workplace policies related to influenza vaccination, we used the following definitions: (1) Free vaccination was defined as the hospitals or local government paying the vaccine and related service costs. (2) Promotion of vaccination was defined as an employer recommending vaccination through a health communication campaign at a facility level, and (3) requirement for vaccination was defined as a workplace issuing official documents requiring vaccination of HCWs.

For the purposes of comparing our study population to that of the general population of HCWs in China, demographic data on the population of HCWs in China were acquired from the National Health Commission of China in 2018 [10]. Gross domestic product (GDP) per capita of each province was obtained from National Bureau of Statistics in 2018 [11].

### 2.3. Statistical Analysis

Influenza vaccination coverage was calculated by dividing the total number of HCWs completing the questionnaires by the number of HCWs reporting vaccine receipt among them. To compare demographic and occupational characteristics, we used Chi-square tests or Fisher exact tests for the categorical variables. Two-sided *p*-value of <0.05 was considered statistically significant. Multivariate logistic regression analysis were used to assess factors associated with influenza vaccination. Demographic and occupational characteristics, workplace vaccination policies were included in the regression model as independent variables.

As the prevalence of vaccination was expected to be higher than 10%, the odds ratio (OR) might overestimate the effects of promotion factors compared to prevalence ratios (PR). We calculated PR based on OR using function: $\mathrm{PR}=\frac{\mathrm{OR}}{\left(1-P_{0}\right)+\left(P_{0}\times \mathrm{OR}\right)}$ to avoid overestimation [12]. In this function, P_0_ means the prevalence of vaccination of HCWs without exposure.

Additionally, a new variable was created combing free policy and requirement/promotion. This new variable was a categorical variable with six classes, namely free policy with requirement/promotion/none, no free policy with requirement/promotion/none. Also, multivariate logistic regression analysis were used to assess this new factor with all the other variables as covariables and PR was calculated.

### 2.4. Human Subjects Review

The study protocol and questionnaire were approved by the ethical review committee at the Chinese Center for Disease Control and Prevention (No. 201901, China CDC, Beijing, China).

## 3. Results

### 3.1. Demographics of Study Population

In total, we sent 116,372 invitations to HCWs, of those, 4,370 opened the survey and 4161 responded. The response rate was 3.6% (4161/116,372). Of the HCWs responded, 4078 had completed responses to all questions in the questionnaire, and we used this number as our study population.

The HCWs surveyed covered 2734 hospitals and 31 provinces in mainland China. Of the 4078 HCWs surveyed, 3316 were physicians, 540 were nurses, 178 were HCWs of other clinical professions (mainly radiologists) and 44 were nonclinical personnel. Age, years of work, and job title of the surveyed HCWs were summarized in Table 1, compared with the whole population of HCWs in China.

The median age of HCWs surveyed was 37 years (ranged from 18 to 79), the median duration of working was 12 years (ranged from less than 1 to 54). In comparison with the population of HCWs in China, HCWs in this study were older, had longer years of work and higher job title (Table 1).

### 3.2. Implementation of Free Vaccination Policy

Among 4078 respondents, 19.0% (774/4078) reported hospital offered free vaccination, 35.4% (1445/4078) reported hospitals promoted influenza vaccination, and 5.4% (221/4078) reported hospitals required HCWs for vaccination.

### 3.3. Influenza Vaccination Coverage among HCWs and Associated Factors

Among the 4078 HCWs, 472 reported they had received influenza vaccine since September 2018. The overall influenza vaccination coverage among HCWs responding to the survey for 2018/2019 season was 11.6%. HCWs provided with free vaccination policy (32.3%) had higher vaccine coverage compared with no free policy (6.7%) (*p* < 0.001). HCWs with workplaces’ requirement (37.6%) or promotion (16.8%) for vaccination had higher vaccine coverage compared with HCWs without workplaces’ requirement or promotion (6.1%) (*p* < 0.001). HCWs working in “high risk departments” had higher vaccine coverage (16.3%) than those not working in high risk departments (10.2%) (*p* < 0.001). Among three levels of hospitals, vaccine coverage was highest among primary care (15.1%), followed by tertiary care (11.5%) and secondary care (10.3%) (*p* = 0.032). HCWs working ≥3 years (11.8%) were more likely to be vaccinated than those working <3 years (5.8%) (*p* = 0.014). In addition, HCWs with senior title (12.2%) and middle title (12.7%) had significantly higher vaccine coverage compared with those with primary and lower title (9.2%) (*p* = 0.013) (Table 2).

Multivariate logistic regression analysis was used to assess factors associated with influenza vaccination and prevalence ratio (PR) was calculated to avoid overestimation of promotion effect using odds ratio (OR). Those who were provided free vaccination by their workplace (PR = 3.67, 95% CI: 3.07–4.35), required (PR = 3.21, 95% CI: 2.48–4.04) or promoted (PR = 1.75, 95% CI: 1.43–2.14) by workplace to receive vaccination, working ≥3 years (PR = 2.09, 95% CI: 1.11–4.03), and working in “higher risk departments” (PR = 1.59, 95% CI: 1.30–1.92) were more likely to get vaccinated (Table 2).

### 3.4. The Impact of Free Vaccination and Workplace’s Vaccination Requirement/Promotion on Vaccine Coverage Rate

The approach of combining free vaccination and workplace’s requirement seemed to be the most effective to improve vaccination rate among HCWs compared with other combinations of these two factors (Table 3). When there was free policy, the vaccination coverage of HCWs was 51.9% with requirement, 32.6% with promotion, and 16.3% without requirement or promotion. When there was no free policy, the vaccination coverage was 15.1% with requirement, 9.2% with promotion, and 5.3% without requirement or promotion.

Multivariate logistic regression analysis also suggested that combing free policy and requirement was the most effective for improving influenza vaccination coverage among HCWs. The prevalence ratio for free policy with requirement was 6.90 (95% CI: 6.03–7.65), free policy with promotion 5.06 (95% CI: 4.30–5.84), free policy without requirement or promotion 2.80 (95% CI: 1.99–3.70), no free policy with requirement 2.48 (95% CI: 1.45–3.68), and no free policy with promotion 1.61 (95% CI: 1.27–2.02) compared with no free policy without requirement or promotion.

### 3.5. Reasons for Vaccination Receipt/Non-Receipt in the 2018/2019 Season

Among the 472 respondents who had received influenza vaccine during the 2018/2019 season, 85.0% believed that the influenza vaccine offered personal protection, 72.3% believed that vaccination can protect their family members. Only 25.2% of HCWs reported that their decision to take vaccine was due to their workplace’s requirement and 22.67% was due to free vaccination. (Table 4).

The main reason HCWs reported for not receiving influenza vaccine was that they were too busy to get vaccinated (51.4%). Other important reasons for not receiving vaccination included a belief that influenza does not cause severe illness (34.7%), do not want to pay for vaccination (24.1%), do not know where to get vaccinated (22.4%), believing that the influenza vaccine was not effective (20.4%), being worried about the side effects (20.3%), and considering that influenza could be easily treated with medicine (12.5%) (Table 5).

## 4. Discussion

The reported influenza vaccination coverage among surveyed HCWs for 2018/2019 season was 11.6% according to this internet-based survey. Only 19.0% HCWs reported their workplace offered free vaccination. In addition, our study reported the free vaccination policy, workplace requirement or promotion, working in “high risk departments” and working ≥3 years were associated with higher vaccination coverage among HCWs. The factor of combing free policy and workplace requirement had the greatest effect on increasing vaccine coverage (PR = 6.90, 95% CI: 6.03–7.65). The main barriers to vaccination were too busy to get vaccinated (51.4%), a belief that influenza did not cause severe illness (34.7%) and do not want to pay for vaccination (24.1%).

Vaccination coverage was 11.6% for HCWs surveyed, this vaccination rate was similar with previous studies conducted in some cities of China [13]. HCWs are one of the recommended groups for influenza vaccine by WHO and China CDC. In October 2018, China’s National Health Commission issued an official document, requiring all hospitals to provide free influenza vaccination for HCWs. The vaccination coverage was much lower than that in the United States (78.4%, 2017–2018) [14]. This difference was probably associated with different vaccination policies in China and the US. Influenza vaccine is generally paid for out-of-pocket in China and there is no immunization program for influenza nationally. While in the US, a large part of hospitals mandate influenza vaccinations for HCWs (61.4% in 2017) [15], along with other approaches that include education efforts, easy access to vaccines, vaccine promotion, etc.

This study reported that influenza vaccination uptake of HCWs was significantly associated with being provided with free vaccination (PR = 3.67, 95% CI: 3.07–4.35) by workplace. This result was in accordance with previous studies in other countries, irrespective of countries’ development status [16,17]. In addition, the study reported a highest vaccination rate (51.9%) among HCWs who were provided with free vaccination and required for vaccination, however, the rate was suboptimal (15.1%) if HCWs were required but no free vaccine was provided. Furthermore, 24.1% HCWs who did not take vaccination reported the reason for non-receipt was they did not want to pay for vaccination. These results supported the approach of providing free vaccination as the core component among all interventions to increase influenza vaccine coverage among HCWs [18]. Providing free vaccination for HCWs was required in the document issued by China’s National Health Commission in 2018 for the first time. Before that, only few cities in China developed reimbursement policy for influenza vaccination for children and old people [3]. However, there was no clear guidance on its implementation or enforcement for this free vaccination policy at the healthcare facility level. We found that only 19.0% (774/4078) HCWs reported that they were provided with free vaccination from workplace. It seems that free vaccination policy was not implemented well in large parts of hospitals in 2018/2019 season. Further evaluations will be needed to monitor the effects of the policy in the future.

Our study reported that influenza vaccination uptake of HCWs was significantly associated with workplace requirement (PR = 3.21, 95% CI: 2.48–4.04) or promotion for influenza vaccination (PR = 1.75, 95% CI: 1.43–2.14) by workplace. This result was in accordance with previous studies [19]. As mentioned above, combining workplace requirement and free policy seems to be the most effective method for improving influenza vaccination coverage in HCWs. Free vaccination policy is important, but we can see that when there was just free policy without requirement or promotion by workplace, the vaccination coverage was only 16.3%, much lower that 51.9% when combing with workplace requirement and 32.6% with promotion. A multi-faceted strategy that include free policy, workplace requirement and promotion, and monitoring should be considered in the future when made guidance on free policy implementation or enforcement [20].

The main reason HCWs reported for not receiving influenza vaccine was that they were too busy to get vaccinated (51.39%), and do not know where to get vaccinated (22.4%). This result indicated that providing on-site vaccination for HCWs may help to improve vaccine coverage.

Our study finding supported that misconceptions of influenza and influenza vaccine could act as a barrier to higher coverage of influenza vaccination [21]. Among 3606 unvaccinated HCWs, 34.7% thought that influenza did not cause severe illness, 20.4% believed that the influenza vaccine was not effective, and 12.5% considered that influenza could be easily treated with medicine. This study reminded the importance of strengthening health communication on the risks of influenza infection and benefits of influenza vaccination among HCWs.

This study has several limitations. Firstly, the HCWs surveyed were sampled from DXY forum, which has about 2.3 million registered HCWs, representing 20% of all the HCWs in China. This population is different from the whole population of HCWs in China, this might result in selection bias. The sample population was older, had longer years of work and higher job title, which might result in a higher evaluation of vaccination coverage according to this study. Secondly, the response rate of this online survey was only 3.6%, the respondents may be different from those who did not respond. There may be response bias due to this low response rate. To encourage participation in future studies, we suggest to take activity of forum users into account when delivering questionnaires, because users with low activity may not see the questionnaire at all. Meanwhile, rewards for participants may be useful, too. Thirdly, we took a brief internet-based questionnaire in this survey, thus the number of questions was limited. We did not collect information about the previous history of influenza, knowledge of influenza and influenza vaccination, perception of vaccine efficacy and side-effects or the history of vaccination so there might be confounding factors for vaccination receipt that were not investigated.

## 5. Conclusions

The current influenza vaccination coverage among HCWs was low in China. To increase future vaccination uptake, we recommend providing free vaccination for HCWs combined with workplace requirement or promotion, on-site vaccination, improving health communication on the risks of influenza infection and benefits of influenza vaccination among HCWs in China. Further evaluations will be needed to monitor the effects of the policy in the future and should focus on the effect of different interventions such as free policy, on-site vaccination and mandatory vaccination on influenza vaccine receipt among HCWs.

## Figures and Tables

**Table 1:** Characteristics of healthcare workers surveyed and in China Health Statistics Yearbook 2018.

Characteristic	Category	HCWs in China (%)	HCWs in This Study (%)	*p*-Value for Chi-Square Test
Age in years	<25	7.9	1.9	0.044
	25–34	38.1	34.9	
	35–44	26.1	41.9	
	45–54	18.6	18.3	
	55–59	3.8	2.1	
	≥60	5.4	0.8	
Years of working	<5	22.7	12.2	0.031
	5–9	23.0	22.7	
	10–19	21.3	36.0	
	20–29	19.2	23.5	
	≥30	13.7	5.7	
Job title	Senior	1.8	6.4	<0.001
	Associate-senior	6.0	23.3	
	Middle	19.6	42.0	
	Primary	29.3	24.9	
	Unclassified/unknown	43.2	3.5	

**Table 2:** Percentage of healthcare workers who received influenza vaccination- Internet panel survey, China, 2018–19 season.

Characteristic	Category	N	Vaccinated	Coverage (%)	*p*	PR (95% CI) ^d^
Occupation	Physicians	3316	398	12.0	0.305	Ref
	Nurses	540	54	10.0		0.8 5 (0.62–1.14)
	Other clinical professionals ^a^	178	15	8.4		0.81 (0.46–1.30)
	Administrative/nonclinical support staff	44	5	11.4		0.84 (0.28–1.85)
Job title	Junior	1158	107	9.2	0.013	Ref
	Middle	1711	217	12.7		1.29 (0.98–1.71)
	Senior	1209	148	12.2		1.17 (0.84–1.61)
Age in years	<30	578	53	9.2	0.140	Ref
	30–44	2632	318	12.1		0.79 (0.53–1.17)
	≥45	868	101	11.6		0.66 (0.40–1.01)
Years of working	<3	174	10	5.8	0.014	Ref
	≥3	3904	462	11.8		2.09 (1.11–4.03)
Hospital level	Unclassified	174	18	10.3	0.032	Ref
	Primary	536	81	15.1		1.50 (0.92–2.43)
	Secondary	1188	122	10.3		1.01 (0.62–1.66)
	Tertiary	2180	251	11.5		0.97 (0.60–1.60)
Working department	Others	3155	322	10.2	<0.001	Ref
	“High risk department” ^b^	923	150	16.3		1.59 (1.30–1.92)
GDP per capita ^c^	Low	734	76	10.4	0.049	Ref
	Middle	1193	122	10.2		0.91 (0.68–1.21)
	High	2151	274	12.7		1.00 (0.77–1.29)
Hospital’s intervention approach	None	2412	147	6.1	<0.001	Ref
	Promotion	1445	242	16.8		1.75 (1.43–2.14)
	Requirement	221	83	37.6		3.21 (2.48–4.04)
Hospital providing free vaccine	No	3304	222	6.7	<0.001	Ref
	Yes	774	250	32.3		3.67 (3.07–4.35)

^a^ Other clinical professionals include: Radiologist, pharmacist, and medical technician. ^b^ “More risky departments” include respiration, infectious diseases, emergency, pediatrics, gynecology and obstetrics and geriatrics, according to our definition in the method part. ^c^ The 31 provinces of mainland China were divided into 3 categories according to GDP per capita. “High” for Beijing, Shanghai, Tianjin, Jiangsu, Zhejiang, Fujian, Guangdong, Shandong, Inner Mongolia and Chongqing; “Middle” for Hubei, Shaanxi, Jilin, Liaoning, Ningxia, Hunan, Hainan, Hebei, Henan, Jiangxi and Xinjiang; “Low” for Sichuan, Qinghai, Anhui, Heilongjiang, Guangxi, Shanxi, Xizang, Guizhou, Yunnan and Gansu. ^d^ Variables included in the multivariable logistic regression include: occupation, job title, age, years of working, hospital level, working department, GDP per capita, hospital’s intervention approach, and hospital providing free vaccine.

**Table 3:** Influenza vaccine coverage in healthcare workers by policy options in 2018–2019 season.

Free Vaccination	Workplace’s Policy	N	Vaccinated	Coverage (%)	PR (95% CI) ^a^
Yes	Requirement	135	70	51.9	6.90 (6.03–7.65)
	Promotion	467	152	32.6	5.06 (4.30–5.84)
	None	172	28	16.3	2.80 (1.99–3.70)
No	Requirement	86	13	15.1	2.48 (1.45–3.68)
	Promotion	978	90	9.2	1.61 (1.27–2.02)
	None	2240	119	5.3	Ref

^a^ Variables included in the multivariable logistic regression include: occupation, job title, age, years of working, hospital level, working department, GDP per capita, combination of hospital’s intervention approach and hospital providing free vaccine.

**Table 4:** Reasons for influenza vaccination receipt- Internet panel survey, China, 2018–2019 season (N = 472).

Reasons for Vaccination ^a^	N	%
Protect myself	401	84.96
Protect my family	341	72.25
Protect patients or persons I cared for	229	48.52
Avoid my work capacity being effected by sickness	215	45.55
Be required by workplace	119	25.21
Have free vaccination offered	107	22.67

^a^ These reasons are not mutually exclusive.

**Table 5:** Reasons for Influenza vaccination non-receipt-Internet panel survey, China, 2018–2019 season (N = 3606).

Reasons for Not Getting Vaccinated ^a^	N	%
I am too busy to get vaccinated	1853	51.39
Influenza does not cause severe illness	1251	34.69
Do not want to pay for vaccination	868	24.07
I do not know where I can get vaccinated	806	22.35
The protecting effect of influenza vaccine is not good	736	20.41
I am worried about the side effects	733	20.33
Influenza can be easily treated with medicine	451	12.51

^a^ These reasons are not mutually exclusive.
